# A qualitative study of barriers to antimicrobial stewardship in Indonesian hospitals: governance, competing interests, cost, and structural vulnerability

**DOI:** 10.1186/s13756-022-01126-7

**Published:** 2022-06-14

**Authors:** Ralalicia Limato, Alex Broom, Erni J. Nelwan, Raph L. Hamers

**Affiliations:** 1 Eijkman-Oxford Clinical Research Unit, Jl. Diponegoro No. 69, Jakarta, 10430 Indonesia; 2 Centre for Tropical Medicine and Global Health, Nuffield Department of Medicine, University of Oxford, New Richards Building, Old Road Campus, Roosevelt Drive, Oxford, OX3 7LG UK; 3 Sydney Centre for Healthy Societies, School of Social and Political Sciences, The University of Sydney, Level 3, Social Sciences Building, Sydney, NSW 2006 Australia; 4 Faculty of Medicine, Universitas Indonesia, Jl. Salemba Raya 6, Jakarta, 10430 Indonesia; 5 Division of Infectious Diseases, Department of Internal Medicine, Cipto Mangunkusumo National General Hospital, Jl. P. Diponegoro no 71 RW 5, Jakarta, 10430 Indonesia

**Keywords:** Antimicrobial stewardship, Barriers, Implementation, Hospitals, Indonesia

## Abstract

**Background:**

Antimicrobial resistance (AMR) is one of the leading global public health threats of the 21st Century. Antimicrobial stewardship (AMS) programmes have been shown to improve antibiotic use and clinical outcomes in high-income settings, but context-specific evidence is lacking on the value and effectiveness of current AMS programmes in low-resource settings. This study sought to explore context-specific underlying barriers to AMS implementation in Indonesian hospitals with a focus on governance practices and structural vulnerabilities.

**Methods:**

We conducted semi-structured interviews with physicians, surgeons, clinical microbiologists, pharmacists, AMS team leaders, hospital managers, medical students, and national AMR stakeholders, and performed a thematic analysis.

**Results:**

Based on 51 interviews conducted between January and October 2020, four main barriers to AMS implementation were evident in the participants' experiences: (1) *Ineffective resourcing* and *institutional buy-in* regarding mandatory AMS under hospital accreditation; (2) *Entangled priorities* to generate profits and interprofessional relationships between doctors and hospital managers or AMS leaders; (3) *Cost-prohibitive bacterial culture testing* and thresholds of national health insurance coverage; (4) *Unreliable infrastructures*, including microbiology laboratory and surgical facilities, ensuring high antibiotic usage to cover structural vulnerabilities.

**Conclusions:**

Limited progress will be made with implementing AMS in Indonesian hospitals, and in settings with similar structural features, without addressing concerns around governance, competing interests, cost and structural vulnerabilities.

## Background

Antimicrobial resistance (AMR) is one of the top ten threats to global public health, with rising antibiotic consumption representing a key driver [1]. A recent global analysis estimated that in 2019 AMR was directly responsible for 1.27 million deaths and played a role in 4.95 million deaths [2]. Global strategies have been developed to preserve the effectiveness of existing antibiotic agents, and in 2015 the WHO launched a Global Action Plan [3]. Institutional action to curb AMR has come largely in the form of antimicrobial stewardship (AMS) programmes [4], aiming to optimizing antibiotic use [5, 6], through tracking and reporting on antibiotic use and resistance, education about resistance and optimal prescribing, and restricting use of particular antibiotics without approval [7, 8]. AMS programmes, predominantly conceptualised in high-income countries [9], have been associated with reducing hospital-acquired infections, unnecessary healthcare costs, and potentially the proliferation of drug-resistant infections in those settings [5, 6]. However, the effectiveness of current AMS models remains unclear for low- and middle-income countries (LMICs) [10, 11] as they may jar with local constraints and practices, and could have limited traction without adequate understanding of the context of implementation [12, 13].

Indonesia is a diverse lower-middle-income country in Southeast Asia with the world’s fourth largest population (273 million) [14]. A range of complex factors, including, but not limited to, persistently high infectious disease burdens, rising antibiotic consumption, a decentralized and fragile health system, and weakly enforced antibiotic policies, have rendered Indonesia particularly vulnerable to AMR [15, 16]. Although hospital AMS programmes in Indonesia were initiated in the early 2000s, their implementation has remained patchy during the first 15 years. Only in 2015, hospitals received a significant push for AMS implementation when the government released regulatory controls [17], and various national guidelines on infection prevention [18], antibiotic use [19], and AMS roll-out [20]. By 2018, AMS programmes were mandatory for hospital accreditation [21]. Despite some progress, those efforts have thus far only produced minor improvements [22, 23]. Many hospitals, as the data provided below further illustrate, inadequately measure process outcomes and structural indicators, e.g., hospital antibiotic guidelines, AMS staff training and education, and human resources [24].

Indonesia has a decentralized public healthcare system, with the Ministry of Health in a coordination role and the provincial and district-level governments having the ownership and authority over their respective hospitals [25]. The private, for-profit, sector also plays an important role in healthcare provision, providing alternatives to depleted public healthcare services [25, 26]. In 2020, the fraction of private hospitals (63.4%) was double that of public hospitals (36.6%), and most laboratories (83%) (including for microbiology) were privately owned [27]. To improve equity of healthcare access [28], in 2014 the Government introduced national health insurance (*Jaminan Kesehatan Nasional, JKN*) [29], and by 2021, 84% of the population had JKN coverage [30], which has led to reductions in out-of-pocket health expenditure [31]. JKN is mandatory for public hospitals [29], while private hospitals can request a contract with the JKN agency [32]. JKN applies prospective fixed claim payment based on diagnosis-related groups [28].

To better understand the context of AMS implementation in Indonesian hospitals, drawing on several private and public hospitals as case studies, in this paper, we begin to identify key underlying barriers with a focus on governance practices and structural barriers, based on stakeholder interviews with a range of medical professionals and policy makers.

## Methods

### Study design

This study was part of the EXPLAIN study that assessed patterns and quality of antibiotic use and potential targets for stewardship intervention in six hospitals in Jakarta, capital of Indonesia [33]. We report here qualitative results based on 51 semi-structured interviews (interviews) conducted between January and October 2020. We also report contextual information on hospital characteristics and stewardship programmes that we collected prior to the interviews.

### Study setting

The participant hospitals were purposively sampled to achieve diversity in geographic location, size, health sector (public and private) and health care level (tertiary and secondary). Participant hospitals were pragmatically selected based on existing collaborations, site willingness, and available study resources. We included two tertiary-care government hospitals and four secondary hospitals, three of which were private hospitals, with between 134 and 853 inpatient beds, and situated across four of the five administrative cities of Jakarta.

At the time of data collection, all six hospitals had an AMS programme in place with a designated coordinating team or committee, albeit at various stages of implementation and time since inception (between 2009 and 2018). One private hospital was only at the planning stage of their AMS programme at the time of the study. All hospitals shared antibiotic guidelines and antibiogram to the prescribers; three hospitals had also implemented education strategies. All public hospitals (3 of 6) employed pre-approval restriction for certain broad-spectrum antibiotics, and one of those also had a weekly AMS round to retrospectively review selected prescriptions and provide feedback to prescribers. All hospitals assessed the interventions outcomes regarding antibiotic consumption and appropriateness of antibiotic use, but only one hospital monitored the compliance of antibiotic guidelines and no hospital evaluated patient clinical outcomes. Most (5 of 6) hospitals did not have a dedicated budget.

### Participants

We targeted varied study participants to obtain different perspectives on the AMS programmes. From each hospital we invited 10–12 participants comprising doctors from medical and surgical departments, a clinical pharmacist, a clinical microbiologist, a representative of hospital management, medical students on clinical rotation, and the AMS team leader. We also invited national AMR stakeholders who were involved in the national AMS programme. We sought variation regarding participants’ stage of career, length of work in their current role, position in the department, and gender.

### Data collection

An interview guide was developed based on the literature and previous research by the author (AB) [34, 35]. The interview process was iterative, and the interviewer prompted follow up questions in line with participant responses. The interviews explored the hospital AMS programme strategies and the perceived factors that influenced implementation. The data collection period was divided into two phases due to the COVID-19 pandemic. Phase 1 comprised face-to-face interviews conducted between January and March 2020 in three hospitals. Phase 2 comprised interviews conducted by video conference between June-July and September–October 2020 in two of three remaining hospitals; one public hospital withdrew its participation due to their high workload during the pandemic.

Depending on the rules of the participant hospital, potential participants were approached by sending an invitation letter through a point-of-contact (POC) who was either the hospital manager, head of department, or AMS team leader. We discussed with the POC about our desired variations in participants’ characteristics as detailed above. Once the participant accepted the invitation and agreed to participate, the POC sent his/her contacts to the interviewer (RL). The interviewer and a research assistant followed-up to arrange the time and venue for the interview. The interviewer had no prior contact with the participants, except with the hospital manager and AMS team leader regarding study administration (hospital permit and interview invitation). On the interview day, before the interview, the interviewer provided information to the participants about the study, the confidentiality, and how the anonymous data would be handled. If the participant agreed, he/she signed a written consent to participate including audio-recording of the interview. The interviews were conducted in Indonesian, audio-recorded, verbatim transcribed and then translated to English. The interviews lasted between 30 and 165 min. Variations in interview duration were mainly determined by the breadth and depth of the discussion, and participants’ time availability. The interviewer also took field notes in all interviews.

### Data analysis

NVivo 12 software augmented data analysis. The five components of the analytical methodology [36] were: (1) data familiarisation; (2) thematic framework identification; (3) data indexing (coding) into themes; (4) charting, rearranging indexed data according to the thematic framework; and (5) data mapping and interpretation. Once themes and codes had been established, the research team members discussed the data interpretation. The initial analysis was performed by RL, which was then cross-checked to facilitate the development of themes by AB, EJN, and RLH. Analytic rigour was enhanced by searching for negative, atypical and conflicting or contradicting cases in coding and theme development. Inter-rater reliability was ensured by integrating several research team members in the final analysis. The interviewer’s notes were used to support the contextual analysis.

## Results

### Participants

A total of 51 participants (23 male and 28 female) were interviewed, including 15 physicians, 11 surgeons, 4 clinical microbiologists, 5 clinical pharmacists, 5 hospital AMS team leaders, 3 hospital managers, 5 medical students, and 3 national AMR stakeholders. Hospital participants were at different stages of their career and had worked for different durations in their respective hospital (< 1 year to > 20 years). The national AMR stakeholders had been in their roles for 2–6 years; two of them also worked in the hospital for > 15 years.

### Barriers to AMS implementation

We found four main barriers to AMS implementation in the hospitals as described by our participants: (1) *Ineffective resourcing* and *institutional buy-in* regarding mandatory AMS under hospital accreditation; (2) *Entangled priorities* to generate profits and interprofessional relationships between doctors and hospital managers or AMS leaders; (3) *Cost-prohibitive bacterial culture testing* and thresholds of national health insurance coverage; (4) *Unreliable infrastructures*, including microbiology laboratory and surgical facilities, ensuring high antibiotic usage to cover structural vulnerabilities.

#### AMS resourcing and institutional buy-in

In 2015, despite national policy [17], as evident in our interviews, AMS was adopted only by few hospitals. AMS programme initiation was challenged at a grassroots level by the highly decentralized health governance system, that is, most public hospitals were under the authority of the regional (provincial and district) governments, whereas there was no policy for AMS implementation at these levels. Therefore, hospitals were not mandated by the regional governments and unable to allocate a budget. This represents a classic gap between mandate and resource, governance and implementation. Similarly, the private hospital participants reported having little coordination with district health officials, and to have not traditionally been partners in public health programmes [26].

To address this complex challenge, the Ministry of Health and national AMR stakeholders made AMS programmes a mandated aspect of hospital accreditation since 2018, with the expectation was that all hospitals would run the programme, including that they would provide dedicated funding.

“…the hospitals didn’t have the authority to provide the funds for the [AMS] implementation because most public hospitals are under regional governments’ authority. They required a black on white [written] regulation that states, ‘this programme must be funded by the local [provincial/district] government’.” (P2 National AMR stakeholder, female)

“We collaborate with the hospital accreditation commission. After two years of lobbying, the accreditation committee was willing to adopt AMS programmes as part of accreditation assessment. The committee made it clear that, “every hospital has to have an AMS team and programme if they want to pass the accreditation with a complete score!” (P1 National AMR stakeholder, male)

At the national level, each of the stakeholders interviewed recognized that programme implementation in most hospitals run ineffectively, only to meet the accreditation criteria.

“Even though an AMS programme is now compulsory because it is evaluated by the hospital accreditation commission, in reality, it ‘only exists’, but the implementation is not as what we were expected. We can see that, ‘oh look, now every hospital has an AMS programme!’ Yes! But the programme has not yet well functioned.” (P2 National AMR stakeholder, female)

“…although it was not stated in the official reports, AMS programmes are executed in full speed only to fulfil the national or international accreditation. But after passing these assessments, management support on the programme decreased. Hospitals implement the AMS programme with a purpose only to being accredited.” (P3 National AMR stakeholder, male)

At the hospital level, hospital manager and AMS leaders/team members mentioned similar issues, i.e., that programme implementation was predominantly pushed for reasons of administrative compliance with accreditation standards.

I: “What is the purpose to form an AMS team in this hospital?”

P: “The first reason is to meet the accreditation standard. Second, we thought that an AMS programme is necessary. But if it had not been forced by accreditation, I think we would not have an AMS team.” (P35 Hospital manager, female)

“Hospital management gives us [AMS team] support in terms of, ‘ok, we provide you with AMS training.’ But the discussion to support this programme was only centred around accreditation. The AMS programme is treated only as a tool to pass the accreditation.” (P18 Clinical microbiologist/AMS team member, female)

#### Funding, priorities, and profit

The expectation that accreditation could enable budget provision for the development of an AMS programme and its effective implementation was met with disappointment, as described in the interviews. Despite the fact that all hospitals had written leadership support, only one had formed an AMS committee and was provided with a dedicated budget. Instead, most hospitals only had an AMS ‘team’ to which no dedicated funding was provided. Incidental funding, for example, to attend trainings and for logistical purposes, were provided to the AMS programme for accreditation interests.

“We, the hospital management, facilitate their [AMS team] needs. But specific funding allocation, like how much money we allocated for the AMS programme, we don’t have that. We provide everything here, so they [AMS team] can use the available resources. For instance, if they want to hold a meeting, the consumption is provided by us.” (P6 Hospital manager, female)

“We [AMS team] are supported by [hospital] regulation because AMS is one of the national programmes. But we do not receive any budget to run the programme because our structure is that of a ‘team’ instead of a ‘committee’. The funding of this programme is tied with other hospital programmes from different departments. For example, to provide AMS trainings [for physicians], we have to tie our programme with the training agenda of the education and training department. We don’t have any freedom to run the programme.” (P12 AMS team leader/infectious disease consultant, female)

In private hospitals, participants recounted how AMS programme implementation was entangled with hospital priorities to generate profits. We noted that pharmaceutical sponsorship for doctors and other healthcare providers has been recognized as a barrier to AMS programmes, and that this practice is common in LMICs where often no strict regulations are applied [37]. In Indonesia, this issue has been regulated in recent years [38], and most participants expressed that healthcare providers are only allowed to receive sponsorship in a form of knowledge/skills building, e.g., for seminars or trainings. However, our participants also noted that conflict of interests endured when hospitals received sponsorship for running some programmes, e.g., health education for patients, and in turn, prevented AMS team running effective programmes to limit antibiotic procurement.

“We can’t deny that the hospital needs funding for its programmes. Once we proposed to stop the antibiotic procurement from a pharma company. Then, we got feedback, ‘we can’t cut off this one. They have sponsored us.’ There are some cases like that, and this complicates things. The expectation is the sponsorship does not affect us, but it still happens even though nowadays it is not as prominent as before. There are many interests involved, not only doctors’ and patients’ interest, but also the hospital management’s.” (P31 AMS team leader/surgeon, female)

Another challenge in private hospitals was the management-doctor relationship. Coherent with studies in other contexts [39, 40], in our setting, specialists/consultants had greater clinical autonomy, owning a status as ‘partners’ rather than subordinates. This influenced the interprofessional relationship, with the AMS team and hospital management apprehensive to enforce the AMS programme as it was perceived as restricting the doctor’s autonomy and created fear that doctors leaving the hospital practice elsewhere.

P: “In this hospital, I cannot interfere in doctors’ decisions. We [pharmacists] were planning to implement an automatic stop order system, de-escalation, and taper dose but it was not approved by the management.”

I: “What was the reason?”

P: “In my view, hospital management cannot patronize doctors’ clinical authority. If we [pharmacists] restrict doctors’ antibiotic prescriptions, maybe the management will receive complaints from them.” (P10 Clinical pharmacist/AMS team member, female)

P: “If we want to execute an ideal stewardship scenario, we must do the pre-prescription authorization approach. But there would be a war and it is not good for doctors’ comfort. We have a mutualism relationship. We need them, they need us –especially the ‘flagship’ physicians.”

I: “What do you mean by that?

P: “The specialists who have many patients. If they left their practice at this hospital, we are left with no patients.” (P6 Hospital manager, female)

Having this complex challenge, as reported by a national AMR stakeholder, some larger private hospitals have re-oriented their AMS programme into a hospital profit-generating scheme.

“I was invited by a private hospital chain. Because this hospital wanted to attract foreign patients, antibiotic use was regulated based on the foreigners’ standard. Westerners are aware that antibiotics are not supposed to be freely prescribed like in Indonesia. This is how they control the antibiotic use in their hospital. Several big private hospitals have already implemented an AMS programme using this strategy.” (P1 National AMR stakeholder, male)

#### Antibiotic susceptibility testing, antibiotic choice, and health insurance coverage

One of the key elements of hospital AMS is diagnostic stewardship, encouraging clinicians to order diagnostic tests, especially bacterial cultures, to tailor the initial empirical antibiotic therapy to a targeted, definitive treatment [41, 42]. In 2020, 2240 hospitals (71.2%) provided health services under the JKN scheme [43]. However, our participants reported that the execution of JKN was experienced as conflicting with diagnostic stewardship. Prescribers considered culture testing to be expensive and exceptional—circa US$ 27–34—while the insurance cover was minimum and variable. Our interviewees reported concerns that insurance would not cover the entire patient management if funds were spent on the diagnostic tests. As such, AMS in practice was deeply connected to the nature of cover, and the regulatory expectations of externalities (in this case, public insurers), rather than hospitals themselves. The normative health insurance did not accommodate diagnostics and thus diagnostic stewardship best practices as part of AMS. Therefore, doctors rely on empiric broad-spectrum antibiotics to cover a range of possible bacterial aetiologies. In some hospitals, local JKN regulations discouraged doctors to order cultures, except for sepsis case or when patients were not improving. This insurance scheme was thus turned into a cost saving scheme.

“When I order a culture [test], it clashes with the JKN system. For example, when a patient is admitted with a urinary tract infection, I immediately order a culture, but the JKN system doesn’t allow me. So, I keep using the empirical antibiotic. When there is no sign of improvement on the third day, by then the culture test request is approved.” (P29 Internist, female)

“There is a challenge, if doctors order cultures in the emergency ward, it is not covered by the JKN scheme; it is only allowed when the patient shows signs of sepsis. The base of this argument is that the emergency ward is only for emergency cases and an infection case is only considered an emergency when sepsis signs occur. Therefore, the culture sample is taken later in the ward to prevent the JKN cap is maxed out in the emergency ward.” (P36 Surgeon/head of emergency department, male)

Another issue, as expressed by our participants, was that antibiotic choice was related to availability and cost under the JKN scheme [44]. To some extent, this became a barrier to the AMS programme, especially when the available antibiotics do not match the susceptibility test result. Besides antibiotic choice, the payment system and concern over insurance cover directed hospitals stakeholders to recommend the use of cheaper antibiotics regardless the spectrum.

“Drug varieties in the JKN scheme are limited. To prescribe antibiotics, we refer to the bacterial aetiology and its antibiotic sensitivity. However, often we are forced to use whatever antibiotics is available in the JKN [drug] list. Therefore, the [antibiotic] review of the AMS team becomes useless.” (P12 AMS team leader/infectious disease consultant, female)

“The JKN scheme covers everything, so antibiotic prescription is not a problem. But the problem is that the recommended surgical antibiotic prophylaxis is cefazolin, a first-generation cephalosporin. It costs 40 thousand rupiah [~US$ 3] per vial, while ceftriaxone, a third-generation cephalosporin, only costs 5 thousand rupiah [~35 cents]. The JKN scheme uses package system, right? It means that the hospital tries to save money as much as possible. Because of this system, it is more profitable to spend 5 thousand rupiah on ceftriaxone than 40 thousand rupiah on cefazolin.” (P19 Obstetrician/gynaecologist, male)

These specific situations demonstrate how AMS implementation is shaped by a range of fiscal and governance externalities which can undermine effective antibiotic optimization practices.

#### Enabling AMS infrastructures: microbiology laboratory and surgical facilities

Budget constraints often challenge establishing and maintaining enabling infrastructures for AMS in low-resource settings. In this study, we found that the challenge was not the lack of infrastructure per se, but the lack of funding flows to ensure standardized and high-quality operations to utilize available infrastructures. Predominantly in public hospitals, prescribers expressed challenges related to microbiology testing, including ambiguous culture results due to unstandardized microbiology facilities and procedures, as well as stock-outs of sample containers and antibiotic discs. Surgeons across hospitals expressed distrust of the sterility of surgical facilities and patient management wards. This was used as a justification to prolong the surgical antibiotic prophylaxis [33] over concerns for surgical site infections.

“The blood samples often don’t meet the standard. The laboratory staff often take a blood sample volume that is less than requested. For instance, I asked for 20cc blood sample, sometimes not even 10cc of blood was taken. Maybe that’s why we often get a false-negative result.” (P17 Internal medicine resident, female)

“Not to mention the broken fridge. Yes, the cooler. Once the culture result was odd! So, I asked the lab doctor, and she said ‘Oh, couple days ago the temperature of the cooler was a bit warm.’ I mean, the lab staff have to inform this situation in the culture report. The culture result might be unreliable since the laboratory condition was unstandardized when they performed the test.” (P13 Intensive care consultant, female)

“The prescribed antibiotic in the surgical wards is not for surgical prophylaxis, but prevention to make sure that the patients don’t get infection. It means, in quotation mark, “I don’t trust the sterility in this hospital.” (P15 Surgeon, male)

## Discussion

Given the structural vulnerabilities of many health systems in LMICs [45, 46], it is unsurprising that AMS programmes have had limited uptake and, when implemented, struggled to gain traction. Here, drawing on qualitative interviews with medical professionals and policy makers, we provide more clarity about what actually occurs at the institutional level in the Indonesian context, potentially with important lessons for other similarly resourced environments. The outcomes indicate a considerable spectrum of challenges, including governance, competing interests, cost, and infrastructural vulnerabilities. These provide a nexus of constraints, which delimit the proliferation and effectiveness of AMS in this context. Each of these dimensions are familiar, but the AMS literature as it currently stands does not provide an integrated view and means to address this implementation complexity in practice.

At the level of *governance*, we found that a ‘separation of powers’ between the national and the local meant that a mere choreography of optimisation/stewardship was inevitable, without sufficient resourcing and commitment. Hospital implementation of AMS was often limited to merely meet the national requirement and driven by accountability structures rather than purpose-centred. This context influenced management to prioritize the administration of regulation vis-à-vis enacting practice changes [47]. Further, AMS was often perceived as an economic burden and not as a revenue generator [48], hence hospitals reported reluctance to provide dedicated funding. To effectively respond to this challenge, there is a need for revised economic models driving AMS programmes by having a clear ‘business’ plan, including programme mapping, clear roles and responsibility of the core team, and an allocated budget [48, 49]. This model would allow AMS teams to work with the available local resources to decide on feasible interventions and measurable clinical outcomes, and to regularly monitor AMS output indicators [50, 51].

At the level of *cost and reimbursement*, the study results illustrate that meaningful AMS, as it currently is conceived, would come at the cost of other necessities, positioning meaningful AMS as not able to be accommodated in reasonable care for the person (i.e., as a part of routine hospital practice and care). Cost saving strategies including postponing culture testing and opting for cheaper antibiotics regardless the spectrum, followed the ongoing low reimbursement provided for care from the JKN scheme. A study exploring prescribing practices under JKN found that physicians and pharmacists practiced ‘rationing’ strategies when the patient medical cost exceeded the JKN tariffs. These included replacing the medicine with less effective ones, reducing the amount of medicine, and encouraging patients to pay for the medicine [52]. Evidence shows that diagnostic stewardship, culture-guided antibiotic treatment [53, 54] and prophylaxis [55], increased cost-effectiveness i.e., decreased treatment and hospitalization expenditures [53, 54]. However, the hospitals in this study did not monitor antibiotic and infection related expenditures, providing no rationale for either investing in AMS or asking for additional resources. Therefore, as found in another study [52], there are clear gaps in cost-effective analysis of the patient management expenditure under JKN, in this case, diagnostic stewardship.

*Vested interests*, especially in private hospitals, provided further energy behind continued over-use, working against de-escalation and illustrating the power of enduring commercial ties in this context (and perhaps others). In LMICs [37, 56], including Indonesia [38], policies to prohibit pharmaceutical sponsorship for commercial purposes are in place. And yet, monitoring and regulatory enforcement of such practice is often weak [37, 57]. Contextualized strategies are needed to develop a stewardship ‘model’ that can work in a particular setting without jeopardizing the quality of care. An alternative strategy observed in this study could be to shift the revenue generation from antibiotic purchase to attracting different patient categories by offering an ideal antibiotic prescribing model.

And finally, *vulnerable infrastructure* raised the risks of optimization, and enhanced uncertainty, unravel the effective implementation of AMS in everyday practice. Similar to other studies in LMICs, we found that hospitals faced operational challenges due to fragile hospital infrastructure and diagnostic facilities. The microbiology laboratory, and economic support for these services, is limited or operated at a minimum level [12, 45], resulting in lack of confidence of prescribers to utilize this service. This finding was in line with the results of the quantitative element of this study (as reported elsewhere [33]) where we found that a bacterial culture was taken in less than half (48.8%) of patients prior to starting therapeutic antibiotics, and only 9% received an antibiotic as guided by a culture result. Infection prevention related to sterilization of the built environment and medical equipment is often inadequate, encouraging doctors to prescribe antibiotics defensively [46]. This qualitative finding resonated with the high proportion (76%) of patients who received prolonged (longer than one day) surgical prophylaxis prescribed in our participant hospitals [33]. These under-addressed everyday issues have become important causative factors for antibiotic over-prescribing and barriers to AMS programmes.

The main limitation of this study was its limited applicability to other hospitals or Indonesia at large. The study focused mainly on urban hospitals in the capital city Jakarta, which are potentially better resourced than many other hospitals in Indonesia. Less resourced hospitals might be confronting different priority challenges. Nonetheless, the study sample included a variation of hospital types including size, sector, health care level, with and without JKN scheme, which improved our ability to understand various dynamics in different hospital settings. We also included the national AMR stakeholders who added the perspective of nationwide AMS implementation challenges.

## Conclusions

In conclusion, antimicrobial stewardship has been implemented nationwide across Indonesian hospitals. Enactment of a national policy, supported by mandatory AMS programmes under hospital accreditation were used as strategies to push programme adoption by hospitals. Despite progress, significant change and sustainability of the programme has been challenged by lack of sustainable support from management, profit generation and interprofessional dynamics in private hospitals, limited national health insurance cover, and limited functionality of enabling AMS infrastructures, microbiology laboratory and surgical facilities. Significant impact of current AMS models is likely to be limited as it requires systemic changes in health governance.

## Data Availability

The datasets used and/or analysed during the current study are available from the corresponding author on reasonable request.
